# Structural insights into vesicle amine transport-1 (VAT-1) as a member of the NADPH-dependent quinone oxidoreductase family

**DOI:** 10.1038/s41598-021-81409-y

**Published:** 2021-01-22

**Authors:** Sun-Yong Kim, Tomoyuki Mori, Min Fey Chek, Shunji Furuya, Ken Matsumoto, Taisei Yajima, Toshihiko Ogura, Toshio Hakoshima

**Affiliations:** 1 Structural Biology Laboratory, Nara Institute of Science and Technology, 8916-5 Takayama, Ikoma, Nara 630-0192 Japan; 2 Department of Developmental Neurobiology, Institute of Development, Aging and Cancer, Tohoku University, 4-1 Seiryo, Aoba, Sendai, Miyagi 980-8575 Japan

**Keywords:** Biochemistry, Cell biology, Structural biology

## Abstract

Vesicle amine transport protein-1 (VAT-1) has been implicated in the regulation of vesicular transport, mitochondrial fusion, phospholipid transport and cell migration, and is a potential target of anticancer drugs. Little is known about the molecular function of VAT-1. The amino acid sequence indicates that VAT-1 belongs to the quinone oxidoreductase subfamily, suggesting that VAT-1 may possess enzymatic activity in unknown redox processes. To clarify the molecular function of VAT-1, we determined the three-dimensional structure of human VAT-1 in the free state at 2.3 Å resolution and found that VAT-1 forms a dimer with the conserved NADPH-binding cleft on each protomer. We also determined the structure of VAT-1 in the NADP-bound state at 2.6 Å resolution and found that NADP binds the binding cleft to create a putative active site with the nicotine ring. Substrate screening suggested that VAT-1 possesses oxidoreductase activity against quinones such as 1,2-naphthoquinone and 9,10-phenanthrenequinone.

## Introduction

Vesicle amine transport protein-1 (VAT-1) was originally isolated as a synaptic vesicle membrane protein, abundantly found in cholinergic synaptic vesicles, and suggested to be involved in vesicular transport^1^. VAT-1 was also identified as a mitofusin (MFN)-binding protein that is localized in the cytoplasm, with a small amount associated with mitochondria, and modulates mitochondrial fusion^2,3^. MFNs are dynamin-related GTPases and GTP-binding promotes MFN dimerization for mitochondria tethering in the process of mitochondria fusion^4^. VAT-1 has also been implicated as playing a role in the phosphatidylserine (PS) transport process, which enables mitochondria to receive PS from the endoplasmic reticulum (ER)^5^. Biochemical studies of VAT-1 have suggested zinc ion-simulative NADPH affinity, putative ATPase activity, and calcium dependency^6–8^. It has also been proposed that VAT-1 plays a role in cancer cell motility^9^. Moreover, a recent study indicated that a natural polyenone, neocarzilin A (NCA), produced by *Streptomyces carzinostaticus*, functions as a potent inhibitor of cancer cell motility by targeting VAT-1-controlled pathways^10^. Although these investigations have suggested a variety of VAT-1 molecular functions in cell regulation, amino acid sequence analysis has indicated that VAT-1 belongs to the NAD(P)-dependent quinone oxidoreductase subfamily. A related protein in the subfamily is ζ-crystallin which is commonly found in the lenses of guinea pig and camel, but not in other mammalian animals including human^11,12^. In an enzyme kinetic study, guinea pig ζ-crystallin (33.9% sequence identity with VAT-1) exhibited high specificity towards NADPH cofactor over NADH^13^. In addition, guinea pig ζ-crystallin^13^ and another ζ-crystallin-like homolog in *Saccharomyces cerevisiae*, yeast Zta1^14^ (25.5% sequence identity with VAT-1), exhibited high reactivity toward orthoquinones, such as 1,2-naphthoquinone and 9,10-phenanthrenequinone. Both ζ-crystallin and Zta1 have been classified as enzymes in the group of NADPH:quinone reductase (EC 1.6.5.5)^15^. At present, however, the potential enzymatic function of VAT-1 seems rather incongruous with the previously proposed functions of VAT-1 in cell regulation. Here, we set out to determine the crystal structure of human VAT-1 and investigate the molecular functions based on the three-dimensional structure. With analysis of the crystal structures of free-form VAT-1 and NADP-bound VAT-1 complex and additional enzymatic studies, we revealed that VAT-1 contains a conserved NADPH binding cleft and binds NADPH to function as an oxidoreductase.

## Results

### Structure determination

**Figure 1:**
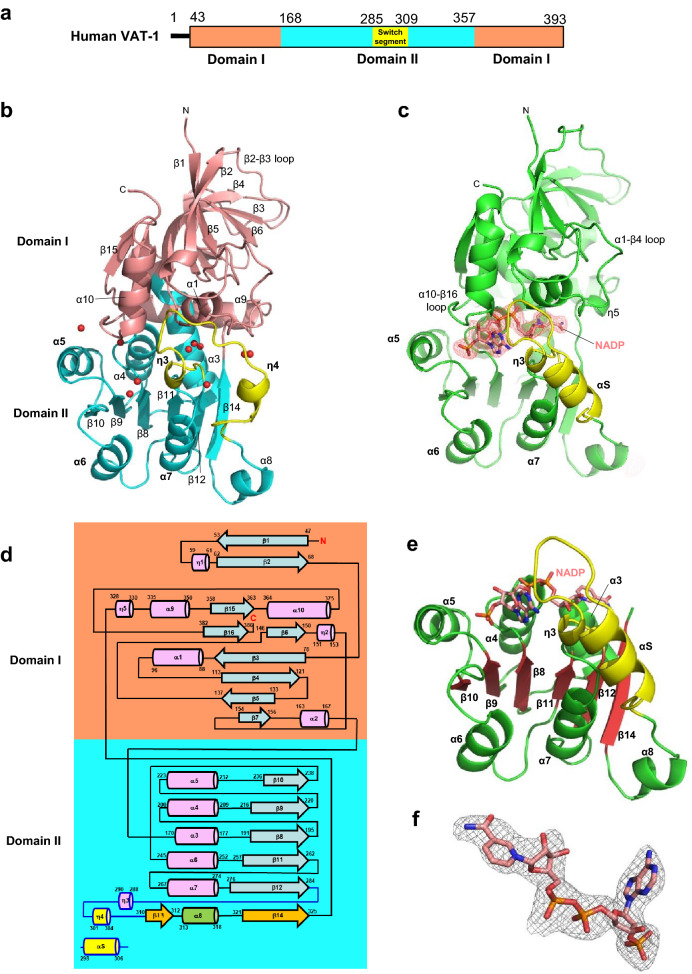
Structure of VAT-1. (**a**) Domain organization of the human VAT-1, which comprised of Domain I and Domain II. A flexible Switch segment was displayed. (**b**) The VAT-1 structure in the free form. VAT-1 comprises two α/β domains, designated as Domain I and II. Water molecules found in the nucleotide-binding site are shown as red spheres. The Switch segment (yellow) covers the binding site. To aid the visualization, α5, α6, and α7 were aligned to NADP-bound VAT-1 in (**c**). (**c**) The VAT-1 structure in the NADP-bound form. The bound NADP molecule is shown as a space-filling model (color codes; carbon in grey, nitrogen in blue and oxygen in red). Large conformational changes are found in the Switch segment (Tyr285–Phe309 in yellow) covering the binding site. Part of the Switch segment is folded into an α-helix (αS). (**d**) Topology of secondary structures found in the free form of VAT-1. Secondary structures comprise α-helices (α1–α10) and 3_10_-helices (η1–η5) (pink cylinders) and β-strands (β1–β16) (blue arrows). Domain I is connected to Domain II by α3–α4 helix and the loop from β14 strand, followed by the η5–α9–β15–α10–β16 segment incorporated in Domain I. Domain II contains the short β–α–β motif for mediating dimerization of VAT-1 that comprises β13 and β14 strands (orange arrows) and α8 helix (green cylinder). The segment between β12 and β13 strands is designated as the Switch segment (yellow) because of its dynamic conformational changes. The Switch segment contains two 3_10_-helices (η3 and η4) in the free form, but in the NADP-bound form one 3_10_-helix (η4) is transformed into a longer α-helix (αS), which is also shown. Residue numbers corresponding to each secondary structure are shown. The six-stranded Rossmann fold β8–α4–β9–α5–β10–α6–β11–α7–β12–α8–β14 provides the fundamental framework for NADPH binding. (**e**) The Rossmann fold in Domain II with the bound NADP molecule found in the crystal structure of the NADP-bound form of VAT-1. The fold comprises β8–α4–β9–α5–β10–α6–β11–α7–β12–α8′ and β14–η5 forming a six-stranded parallel β-sheet with sequence β10–β9–β8–β11–β12–β14. (**f**) The NADP molecule in the bound form of VAT-1 with omit map which was calculated by a refinement of Phenix.refine with NADP-deleted model (contoured at 3σ).

Recombinant human VAT-1 was purified and crystallized. Since use of the full-length (1–393 residues) protein yielded crystals that diffracted only to low resolution (below 8 Å), we sought to generate crystals that could diffract to higher resolution by employing a truncated protein for crystal formation. Truncation of the poorly conserved N-terminal region of the protein to generate N-truncated VAT-1 (43–393 residues), hereafter referred to simply as VAT-1 unless otherwise stated (Fig. 1a, Suppl. Fig. S1), yielded crystals that diffracted to 2.3 Å resolution. The structure was determined by molecular replacement using the known structure of a VAT-1 homolog-like protein (PDB ID 4A27) and refined (Suppl. Table S1). The obtained structure revealed a tunnel that possesses a conserved NADPH-binding motif and is obviously expected to accommodate an NADPH molecule. We confirmed that NADPH binds VAT-1 with low affinity using low-c isothermal titration calorimetry (ITC) method^16^ (Suppl. Fig. S2) and crystallized VAT-1 with bound NADP. We obtained crystals that diffracted to 2.62 Å resolution and the structure was refined (Suppl. Table S1).

### Overall structure

The VAT-1 molecules in the free and NADP-bound states display essentially the same kidney-shaped structures (Fig. 1b,c). VAT-1 comprises ten α-helices (α1–α10), five 3_10_-helices (η1–η5) and sixteen β-strands (β1–β16), and forms two α/β domains, designated as Domain I and II (Fig. 1d). Domain I is formed by 126 N-terminal residues (Ala43–Ala168) and 37 C-terminal residues (His357–Asn393), while Domain II is formed by 187 residues (Leu169–Pro356) (Fig. 1a). Domain I comprises nine β-strands consisting of an isolated two-stranded antiparallel β-sheet (β1–β2), a six-stranded mixed β-sheet (β15–β16–β3–β4–β5–β7), which is highly twisted, and β3-strand forms an additional antiparallel β-sheet with β6-strand. Domain II comprises seven β-strands consisting of a six-stranded parallel β-sheet (β10–β9–β8–β11–β12–β14) and one isolated β-strand (β13). The long segment (25 residues) between β12 and β13 strands is conformationally flexible and displays different conformations among the crystallographically independent molecules in the crystals with part of the segment being disordered (Suppl. Fig. S3). We refer to the β12–β13 segment as the Switch segment (Tyr285–Phe309) because of the dynamic conformational changes that are invoked upon NADP binding (Fig. 1b,c), which will be discussed in detail below.

The VAT-1 structure formed by the two α/β domains is closely related to that displayed by members of the quinone oxidoreductase family (Suppl. Fig. S4). When the VAT-1 structure is superimposed on the starting structure of human VAT-1 homolog-like protein, which exhibits 45.8% sequence identity, the root-mean-square (rms) deviation of the Cα atoms is 1.13 Å. We found that ζ-crystallin-like quinone oxidoreductase Zta1^14^ (PDB ID 3QWB, with sequence identity of 25.5%) displays a closely related structure with a relatively small Cα atom rms deviation of 1.51 Å. CurF ER^17^ from *Lyngbya majuscule* (PDB ID 5DP2, with 26.9% sequence identity) also exhibits a small Cα atom rms deviation of 1.67 Å. It should be noted that Zta1 is an active quinone oxidoreductase and that CurF ER is a unique enoyl-acyl carrier protein reductase that catalyzes cyclopropanation in the curacin A biosynthesis pathway^17^. The similarity in structure between VAT-1 and the aforementioned enzymes led us to investigate the potential enzymatic activity of VAT-1 (full-length) and VAT-1 (43–393) (see below).

### Rossmann fold nucleotide-binding site

The obtained structures revealed that VAT-1 conserves a Rossmann fold^18^ in Domain II with the GXGXXG motif for nucleotide binding, a modified AXGXXG motif, AAGGVG (residues 197–202), at the N-terminal end of α4-helix (Fig. 1e). We observed clear electron density for the bound NADP molecule (Fig. 1f). The nucleotide-binding site is located at the interface between Domain I and II, and is covered by the long 25-residue segment between β12 and β13 strands, extending from Domain II toward Domain I (Fig. 1b,c). The nucleotide-binding site is also partly covered by Domain I, which projects α10-helix, α10–β16 loop, α1-helix and α1–β4 loop toward the binding site. Moreover, η4-helix blocks one edge of the binding site. As a result of being covered by Domain I and the Switch segment from Domain II, the nucleotide binding site is embedded in the interface between Domain I and II and forms a tunnel. In the free form of VAT-1, the tunnel is filled with solvent water molecules (Fig. 1b). In the NADP-bound form, the nucleotide-binding tunnel is filled by the bound NADP molecule, which adopts an extended conformation and is almost completely buried inside (Fig. 1c). Only part of the adenine ring at one entrance and part of the nicotinamide ring containing the amide group are accessible from the solvent region (Suppl. Fig. S5).

### Dimeric structure

**Figure 2:**
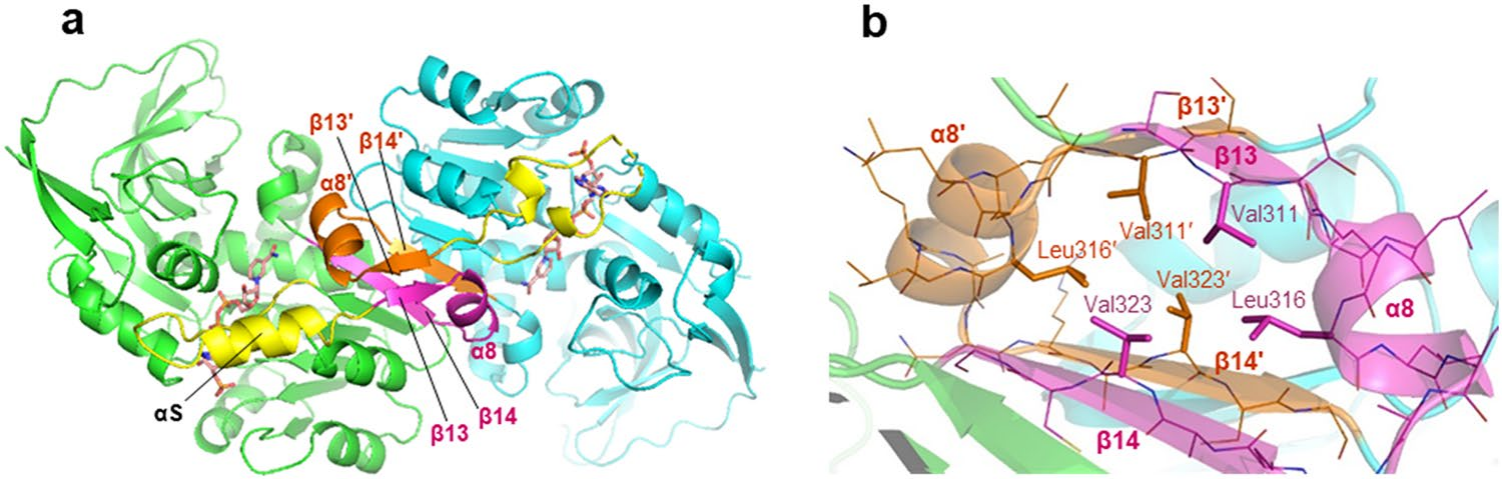
VAT-1 dimer. (**a**) The VAT-1 homodimer in the NADP-bound form. The bound NADP molecules are shown as stick models (orange). The β13–α8–β14 segment of each VAT-1 protomer forms the βαβ motif. The βαβ motif (magenta) of VAT-1 mol A (green) and the βαβ motif (orange) of mol B (cyan) mediates the dimer interface by forming intermolecular antiparallel associations between β13 and β13′ strands, and β14 and β14′ strands. (**b**) The βαβ motifs from two protomers build a compact module by forming a mini hydrophobic core with hydrocarbon side chains from Val311 (at β13 strand), Leu316 (α8 helix) and Val323 (β14 strand).

The obtained crystal structures showed that VAT-1 forms a homodimer in which each protomer is related by a pseudo dyad axis, as observed in the structures of the related oxidoreductase family members^14,17,19^ (Fig. 2a). The dimerization is mediated by Domain II. The β13–α8–β14 segment (the βαβ motif) in Domain II is packed into a compact fold by forming a hydrophobic mini core (Val311–Leu316–Val323) between sandwiched β13 on β14 strands and plays a key role in formation of the dimer interface (Fig. 2b). The βαβ motifs from two protomers form an intermolecular two-stranded antiparallel β-sheet (β13–β13′) and an intermolecular β14–β14′ antiparallel association, resulting in formation of an extended 12-stranded β-sheet from the six-stranded parallel β-sheets of the two protomers. In addition to the β–β association, α8-helix from each protomer forms nonpolar interfaces to mediate the dimerization. The fundamental architecture and dimerization mediated by the βαβ motif are conserved in the NADP-bound VAT-1 structure, though local conformational changes occur upon nucleotide binding as described below.

### NADP binding

**Figure 3:**
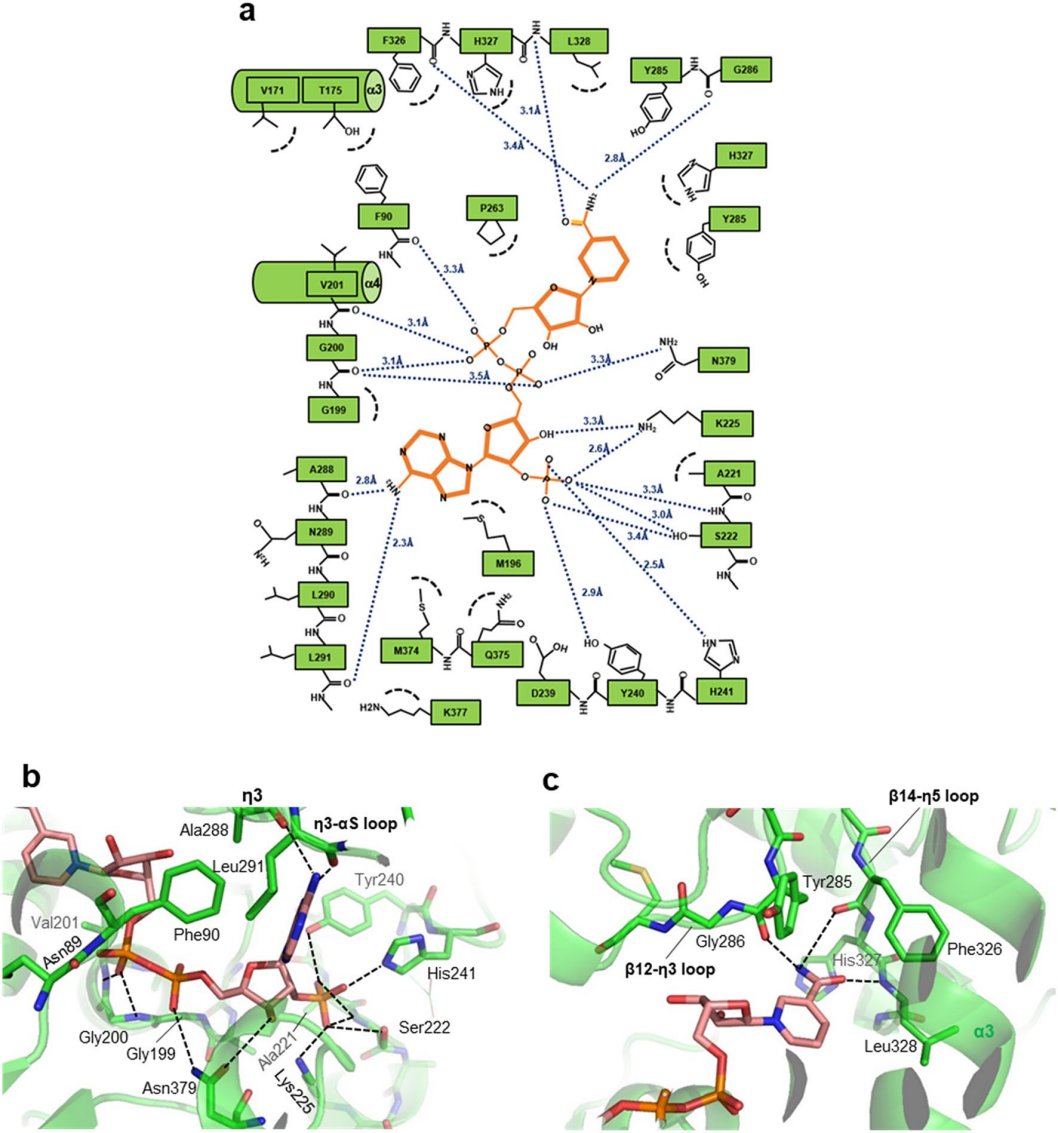
NADP bound to VAT-1. (**a**) Graphical summary of the NADP-VAT-1 interactions calculated by LIGPLOT^33^. (**b**) The ADP moiety of the bound NADP forms 12 hydrogen bonds with VAT-1. (**c**) The NMN moiety of the bound NADP forms 6 hydrogen bonds with VAT-1.

The bound NADP molecule is tightly held in the narrow tunnel of VAT-1 with a fixed conformation without any residual mobility (Fig. 1c). The NADP/NADPH cofactor comprises the ADP (adenosine-2′,5′-diphosphate) moiety and NMN (nicotinamide 5′-mononucleotide) moiety. The bound NADP molecule has the ADP moiety in the *syn* conformation with the C3′-*endo* pucker of the ribose ring conformation and the NMN in the *anti* conformation with the C2′-*endo* pucker of the ribose ring conformation. In the binding tunnel, VAT-1 forms fourteen direct hydrogen bonds with the bound NADP molecule (Fig. 3a).

At one entrance of the tunnel, β9–α5 loop (Ser222), α5-helix (Lys225) and β10–α6 loop (Tyr240, His241) from Domain II form the phosphate-binding site and tightly trap the 2′-phosphate group of ADP moiety by forming six hydrogen bonds with the site, indicating the importance of the 2′-phosphate group of NADPH in VAT-1 binding (Fig. 3b). The adenine ring is recognized by forming two hydrogen bonds between the adenine 2-amino group and the Switch segment (the main chains of Ala288 and Leu291 of η3-helix) from VAT-1 covering the binding site. The 3′-hydroxyl group of the ADP ribose forms a hydrogen bond with conserved Asn379 (at α10–β16 loop) from Domain I. Asn379 also forms a hydrogen bond with the 5′-phosphate of ADP and fixes the ADP conformation by bridging two parts of ADP through two hydrogen bonds. The diphosphate group at the center of the NADP molecule is fixed at the middle of the tunnel by forming five hydrogen bonds with VAT-1. With one of the diphosphate groups, the 5′-phosphate group of the NMN moiety is trapped by the AXGXXG motif at the N-end of α4-helix (Fig. 3c). The nicotinamide ring forms three hydrogen bonds via the amide group of the nicotinamide with the main chains of β14–η5 loop (Phe326 and Leu328) and the N-terminal end of the Switch segment (Tyr285), whereas the ribose of the NMN moiety has no direct hydrogen bond.

Nonpolar interactions also contribute to NADP binding. The adenine ring is sandwiched with Lys377 (at α10–β16 loop) and Phe90 (α1-helix)/Met196 (β8–α4 loop). Phe90 and Met196 also contact with the ribose of the NMN moiety. Thus, Phe90 and Met196 are sandwiched between two ribose rings of the NADP molecule. The nicotinamide ring sits on Tyr285 (Switch segment), Thr175 (α3-helix), Val201 (α4-helix) and Phe326 (β14–η5 loop).

### Switch segment dynamics

**Figure 4:**
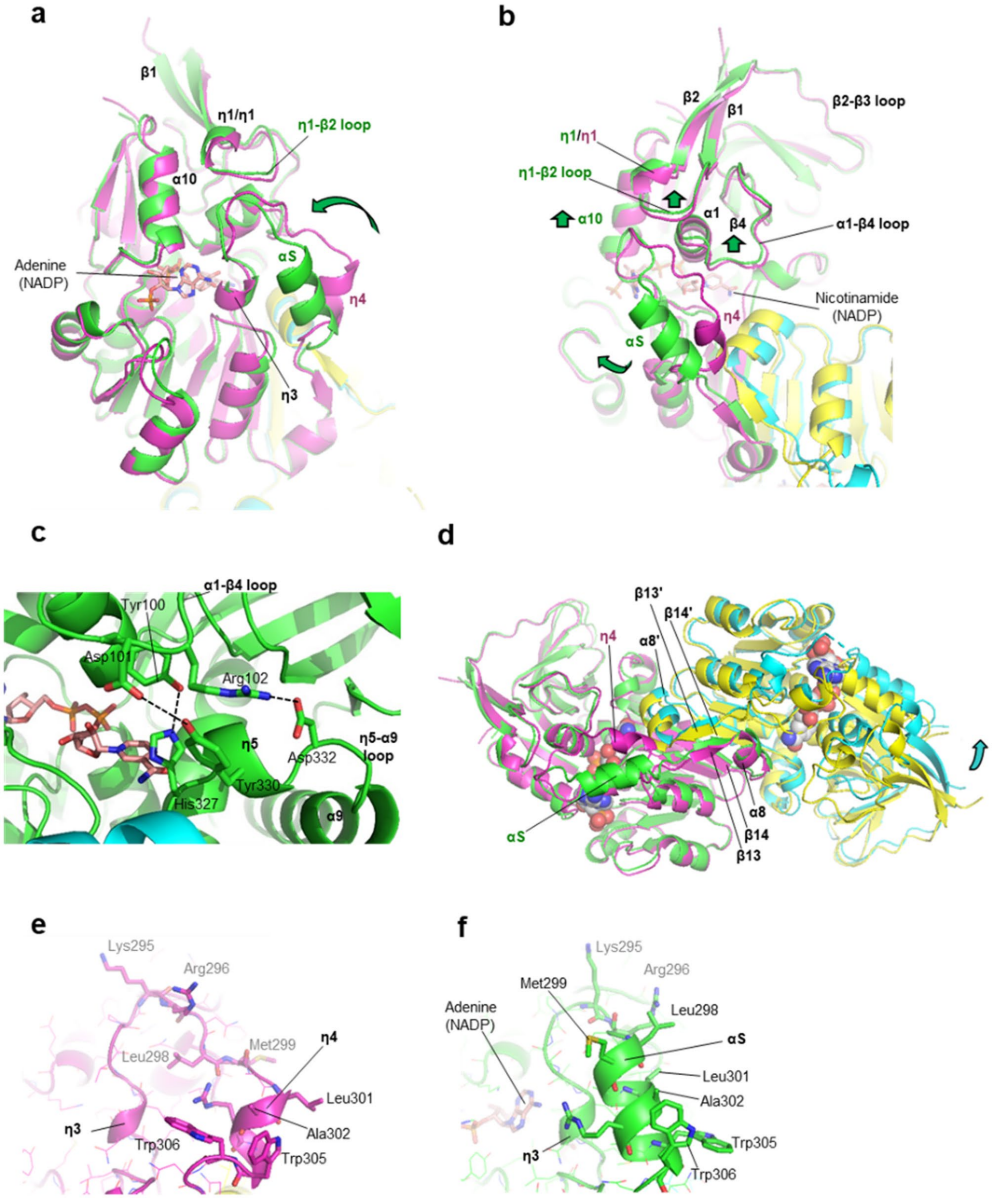
Induced fit and conformational dynamics of the switch loop on NADP binding. (**a**) The induced fit conformational change on NADP binding. Structural overlay is shown for NADP-bound VAT-1 (green) on the free form (magenta). An arrow (green) indicates the large shift of the Switch segment on NADP binding. A view for the 2′,5′-ADP moiety of the bound NADP (stick model) is provided with the dimer partner in the NADP-bound form (cyan) and in the free form (yellow). (**b**) As in (**a**) but a rotated view of 90° around the vertical axis to show a view of the NMN moiety of the bound NADP. Arrows (green) indicate the large shift of the Switch segment and small local shifts of α1–β4 loop, η1–β2 loop and α10-helix on NADP binding. (**c**) Formation of the hydrogen bonding bridge widens the nucleotide-binding site. In the NADP-bound form, α1–β4 loop of Domain I forms hydrogen bond/salt bridge interactions with η5-helix and η5–α9 loop of Domain II. Hydrogen bonding residues comprise Tyr100–His237 and Asp101–Tyr326 and the salt bridge comprises Asp102–Asp332. (**d**) Conformational changes on NADP binding affect the dimer configuration. Structural overlay is shown for the VAT-1 dimer in the NADP-bound form (green and cyan) on the free form (magenta and yellow). The bound NADP molecules are shown as space-filling models. One protomer (green) of the NADP-bound form is superimposed on one protomer (magenta) of the free form to clarify the positional shifts of the other protomers. (**e**) Exposure of nonpolar residues from αS-helix of the Switch segment in the free form of VAT-1. (**f**) Exposure of nonpolar residues from αS-helix of the Switch segment in the NADP-bound form of VAT-1.

NADP binding induces conformational changes in the VAT-1 structure. The major conformational change is found in the Switch segment (Fig. 4a,b). As mentioned above, the Switch segment participates in recognition of both adenine and nicotinamide rings of NADP. In the NADP-bound form, the Switch segment induces conversion of short η4-helix to longer αS-helix and approaches η3-helix to make nonpolar helix-helix contacts.

These interactions should stabilize η3-helix to interact with the adenine ring. In addition to the dynamic conformational change in the Switch segment, helices and loops forming the binding site are also shifted to accommodate the NADP molecule. Among these, α1-helix and the following α1–β4 loop from Domain I is lifted by ~ 1 Å to enlarge the binding tunnel by forming hydrogen bonds with η5-helix and η5–α5 loop from Domain II (Fig. 4c).

The Switch segment is directly linked to β13-strand, suggesting that conformational changes in the Switch segment affect the βαβ motif that mediates dimerization. Moreover, the Switch segment makes direct contacts with α8-helix of the other protomer in the free form (Fig. 4d). On NADP binding, the Switch segment moves from α8-helix of the other protomer, which results in a shift of the protomer (by maximum of ~ 6 Å).

The Switch segment contains clusters of nonpolar residues, being characterized by two tryptophan residues (Trp305 and Trp306). Intriguingly, most of these nonpolar residues are exposed to the solvent region (Fig. 4e). On NADP binding, αS-helix is folded, whereas most of the nonpolar residues are still exposed to the outside (Fig. 4f). These exposed nonpolar residues (Leu298, Met299, Leu301, Ala302, Trp305 and Trp306) participate in crystal contacts with adjacent VAT-1 molecules by making nonpolar contacts with nonpolar residues of the Switch segment (Suppl. Fig. S6). In the crystal of the NADP-bound form, nonpolar contacts between αS-helices were found to include intermolecular tryptophan–tryptophan interactions in the crystals (Suppl. Fig. S6a). These intermolecular interactions may contribute to mediate dimer–dimer interactions in solution. We examined this possibility by analyzing both VAT-1 (full-length) and VAT-1 (43–393) at various concentrations using size-exclusion chromatography (SEC) (Suppl. Fig. S7a,b). Both VAT-1 constructs displayed a similar trend in solution as the proteins tend to form dimer at low concentration and shift to tetramer (dimer–dimer) at higher concentrations. VAT-1 (full-length) was eluted as tetramer peak at the concentration of 300 μM, meanwhile the VAT-1 (43–393) exists as tetramer–dimer equilibrium. The results of SEC are in agreement with the current crystal structures, where VAT-1 exists in dimer and dimer–dimer conformations. The conformation of the Switch segment was affected by the NADP-binding as shown in our crystal structures, we continued to analyze the effect of its binding towards the oligomerization of VAT-1 (Suppl. Fig. S7c,d). In the presence of 10-folds higher molar concentration of NADP, VAT-1 (full-length) remained unaffected as free forms. Surprisingly, a broader shoulder peak was observed for 50 μM VAT-1 (43–393) in the presence of 500 μM NADP, indicating that VAT-1 (43–393) has a higher tendency to shift to dimer by the addition of NADP in comparison to VAT-1 (full-length). However, the exact reason for the reduced tendency in tetramerization exhibited by the truncated VAT-1 (43–393) in the presence of excess NADP was unclear.

### Putative substrate binding site

**Figure 5:**
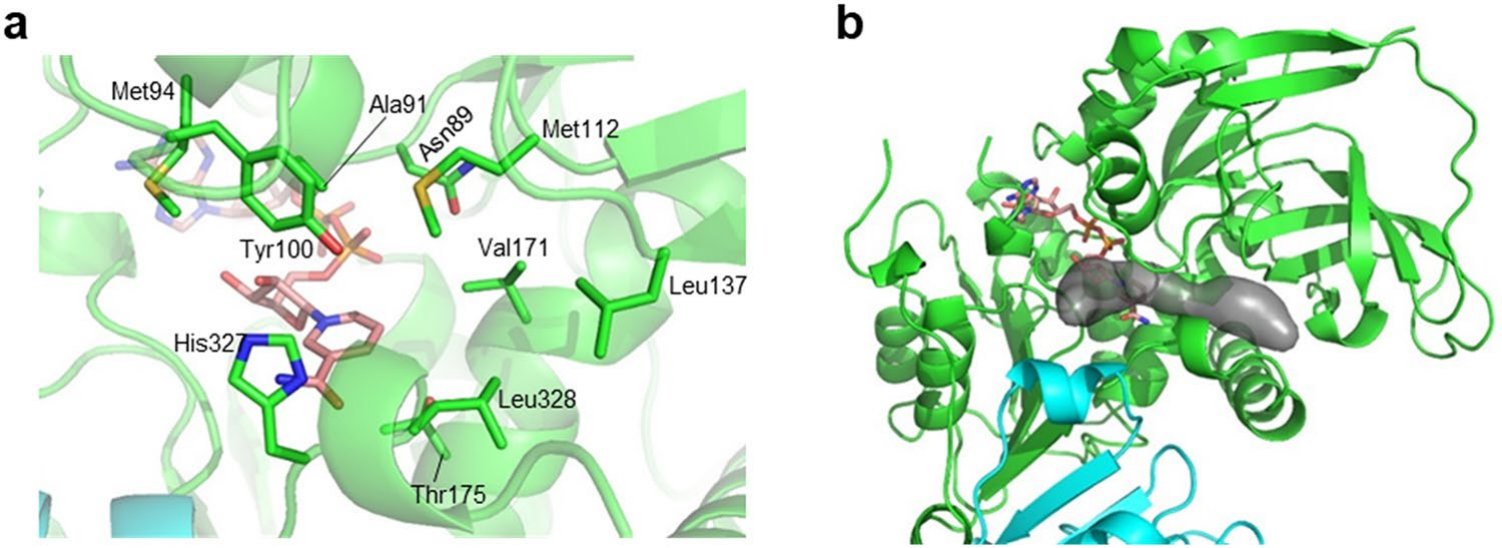
Putative substrate-binding site. (**a**) The putative substrate-binding site on the nicotinamide ring plane. (**b**) The cavity of the putative substrate-binding site is depicted as a gray surface.

Intriguingly, one side of the nicotinamide ring in the nucleotide-binding tunnel is open without any VAT-1 residues making direct contacts with the ring (Fig. 5a). The bound nicotinamide ring should provide the active site of the potential oxidoreductase activity of VAT-1 as with members of the quinone oxidoreductase family^14,17,19^. Zta1 also has a large space on the nicotinamide ring of the bound NADPH molecule and a small glycerol molecule was found to be located at the space^14^. This space is proposed as the substrate binding site of substrates such as 1,2-naphthoquinone (NQ) and 9,10-phenanthrenequinone (PQ). We thought that the large empty space on the nicotinamide ring plane in our NADP-bound VAT-1 structure may be the substrate binding site for potential oxidoreductase activity (Fig. 5b). The space is rather flat and surrounded by nonpolar residues (Ala91, Met94, Met112 and Leu137 from Domain I, Val171 and Leu328 from Domain II) and polar residues (Asn89 and Tyr100 from Domain I, and Thr175 and His327 from Domain II) (Fig. 5a). Interestingly, Asn89 of VAT-1 seems to correspond to Asn48 of Zta1, which was proposed to participate in recognition of substrates NQ and PQ by forming a direct hydrogen bond with the carbonyl group of each substrate^14^. In addition to Asn48, residues Ile50 and Leu131 of Zta1 were shown to contribute to substrate binding. These two residues correspond to Ala91 and Val171 of VAT-1. Therefore, the putative substrate-binding space of VAT-1 may be slightly larger than that of Zta1. As proposed for Zta1, electron transfer via π–π stacking interactions may occur once the phenyl ring of quinone substrate compounds is aligned parallel to the nicotinamide ring. An increase in hydrophobicity around the positively charged nicotinamide group is likely to accelerate electron transfer from NADPH to substrate.

### Quinone oxidoreductase activity

**Figure 6:**
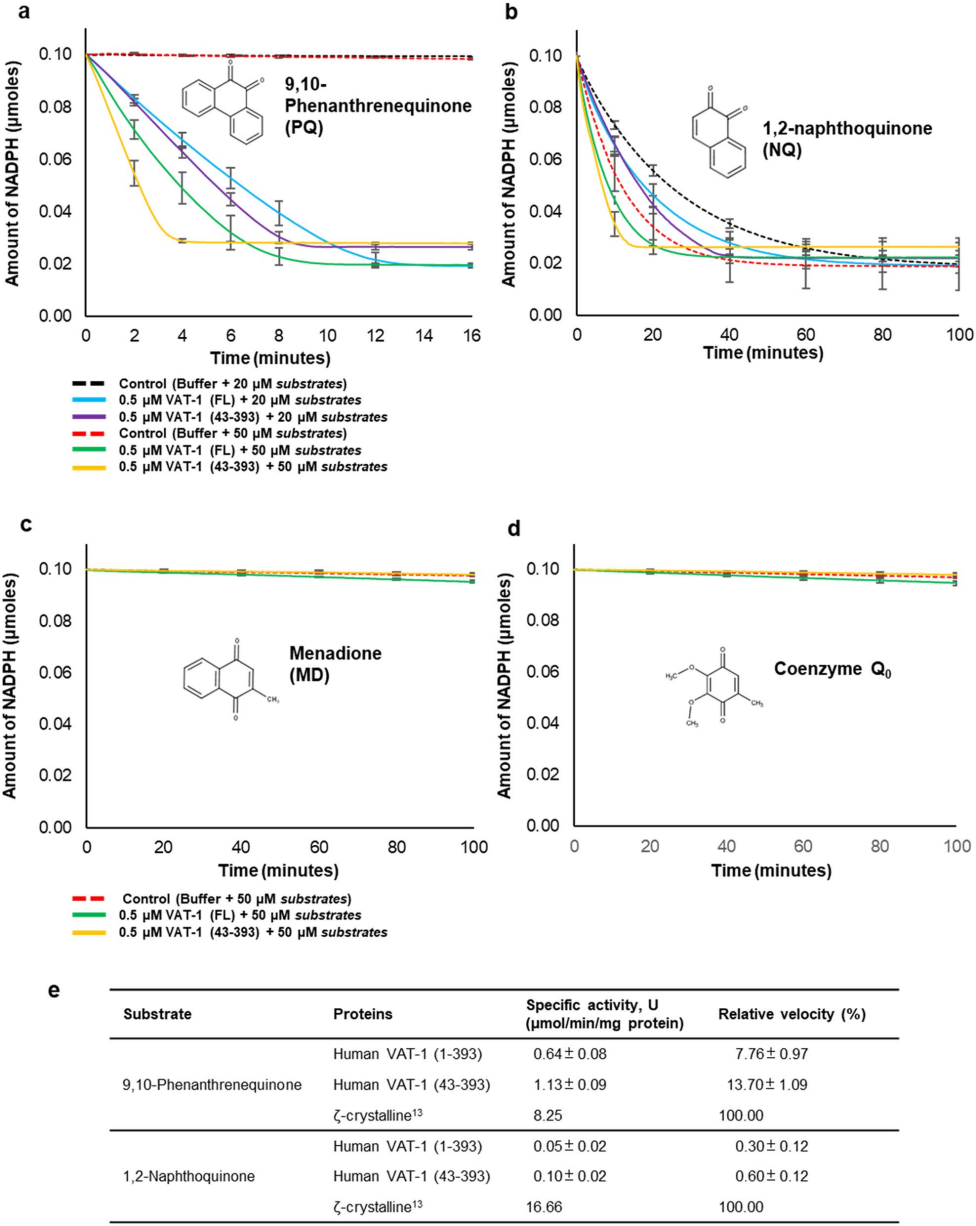
Potential quinone oxidoreductase activity. (**a**) Time-course of NADPH decrease in the reaction of VAT-1 (full-length) and VAT-1 (43–393) with 1,2-naphthoquinone (NQ). The chemical structure is shown in the panel. Absorbance at 340 nm was monitored to detect the decrease of the NADPH in the assay. Reactions were performed in mixtures containing 100 mM Tris–HCl (pH 7.8), 100 μM NADPH, 20 and 50 μM NQ. Control was performed without the addition of VAT-1. All the reactions were performed in triplicates, and the error bars were indicated. (**b**) As in (**a**) but for 9,10-phenanthrenequinone (PQ). (**c**) Time-course of NADPH decrease in the reaction of VAT-1 (full-length) and VAT-1 (43–393) with 50 μM menadione (MD), which is part of vitamin K3. (**d**) As in (**c**) but for ubiquinone 0 (UQ_0_), which is part of coenzyme Q0. (**e**) Comparison of the relative activities of 0.5 μM VAT-1 (full-length)/(43–393) to that of the ζ-crystalline, using 50 μM of 1,2-naphthoquinone and 9,10-phenanthrenequinone as substrates.

**Figure 7:**
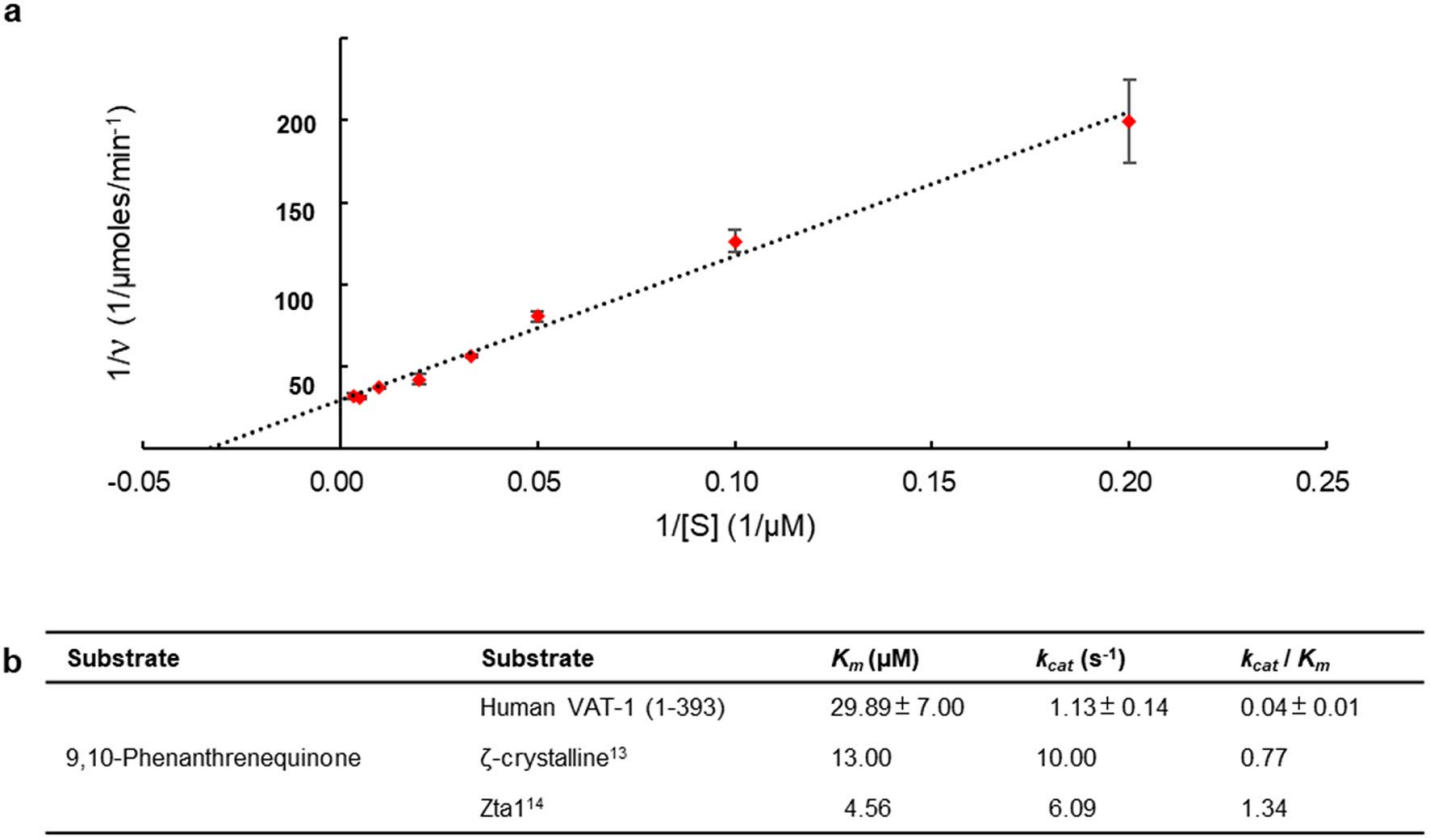
Enzyme kinetics of VAT-1. (**a**) The Lineweaver–Burk reciprocal plot of VAT-1 (full-length) versus PQ. The *K*_m_ of VAT-1 (full-length) towards PQ was calculated as 30 μM. The initial velocities were measured at 5, 10, 20, 30, 50, 100, 200, and 300 μM. All reactions were measured in triplicates. (**b**) The *K*_m_ and *k*_*cat*_ of VAT-1 (full-length) in compared to that of the ζ-crystalline.

Our structure of the NADP-bound form clarified that VAT-1 contains a conserved nucleotide-binding site, which is common to members of the NADPH-dependent oxidoreductase subfamily, and that NADP binds to this site of VAT-1 in a manner similar to the binding of NADP/NADPH to enzymatically active members of the oxidoreductase subfamily such as Zta1. Moreover, we found a putative substrate-binding site on the nicotinamide ring of NADP. These results suggest that VAT-1 may possess oxidoreductase activity against some quinone derivatives. We then investigated the enzymatic activity of VAT-1 against certain potential substrates including 1,2-naphthoquinone (NQ) and 9,10-phenanthrenequinone (PQ), the substrates for Zta1 and ζ-crystallin^13,14^, as well as menadione (MD) and ubiquinone 0 (UQ0) (Fig. 6). MD is vitamin K_3_, while UQ0 is coenzyme Q0. Among these quinones, both VAT-1 (full-length) and VAT-1 (43–393) possessed the highest specificity toward PQ at a specific activity of 0.64 U and 1.13 U, respectively (Fig. 6a,e). VAT-1 (full-length) and VAT-1 (43–393) reacted to NQ at a much slower rate and possessed a specific activity of 0.049 U and 0.102 U, respectively (Fig. 6b,e). Although VAT-1 (full-length) and VAT-1 (43–393) reactive towards PQ and NQ, the activities were relative low, which were 7.8 and 13.7% for PQ, and 0.3 and 0.6% for NQ, respectively, compared to ζ-crystallin^13^ (Fig. 6e). Interestingly, VAT-1 (43–393) consumed PQ and NQ at a higher rate and displayed nearly twofold higher activities over the VAT-1 (full-length). In contrast, both of VAT-1 constructs did not show any significant activities towards MD and UQ0 (Fig. 6c,d). To investigate the kinetic properties of VAT-1 (full-length) towards PQ, we calculated the kinetic constants from double reciprocal Lineweaver–Burk plots of the reversed velocity (1/ν) versus the reversed substrate concentration (1/S) (Fig. 7a). In the presence of PQ at various concentrations, the initial velocities exhibited by 0.5 μM VAT-1 (full-length) were measured and plotted. The calculated *K*_*m*_ and *k*_*cat*_ of VAT-1 (full-length) were 29.89 μM and 1.13 s^−1^ respectively, indicating that VAT-1 (full-length) is an active quinone oxidoreductase (Fig. 7b).

## Discussion

We have demonstrated that VAT-1 has the α/β fold structure exhibiting essentially the same architecture as the NADPH-dependent quinone oxidoreductase structures that are characterized by a kidney-shaped structure comprising two domains in α/β folds, designated as Domain I and II. The interface between Domain I and II form a deep tunnel of the nucleotide-binding site with a conserved sequence for phosphate binding on the Rossmann fold of Domain I. Part of the nucleotide-binding site provides a small pocket for putative substrate binding as observed in other oxidoreductases such as Zta1^14^.

**Figure 8:**
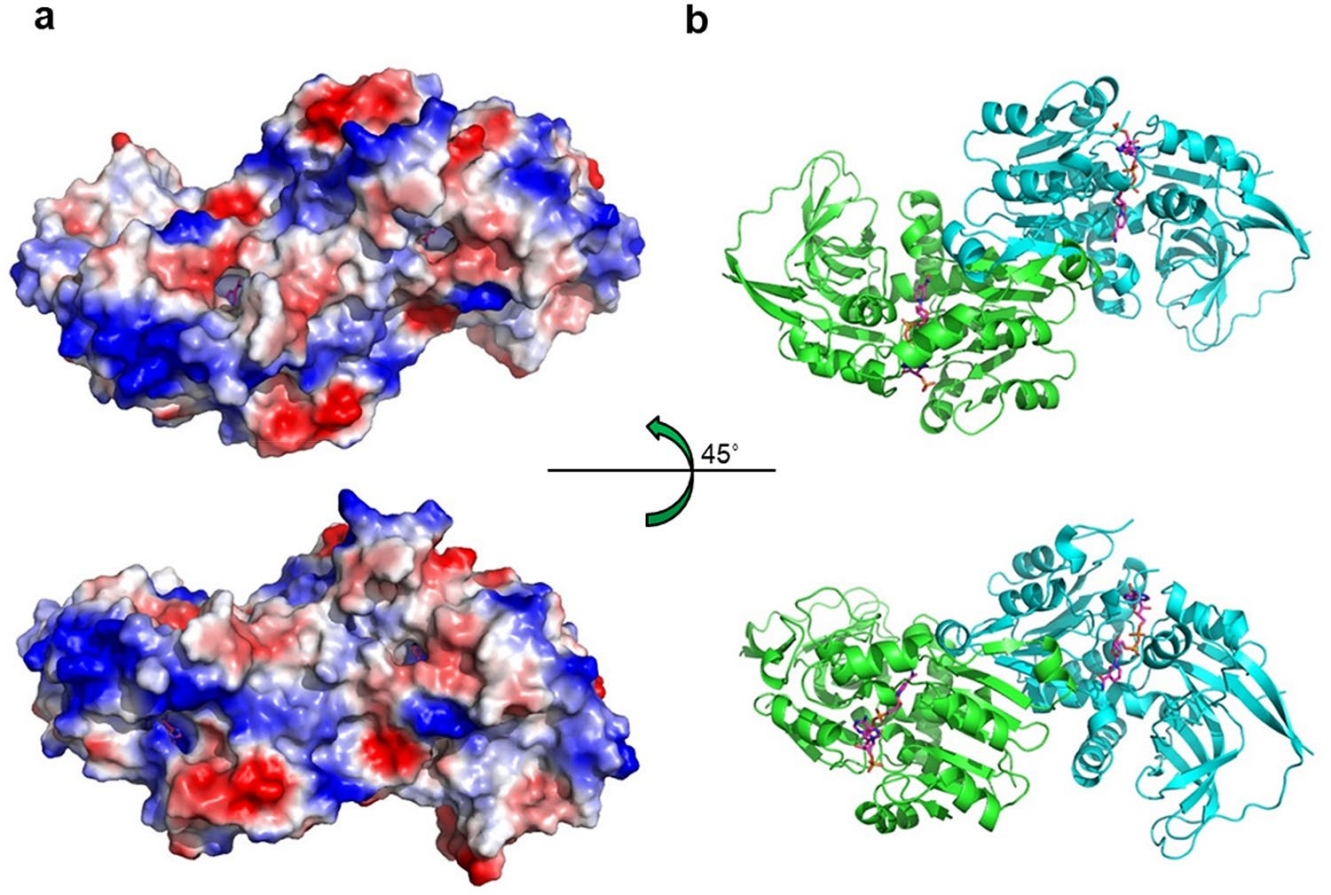
Molecular surface of VAT-1. (**a**) Electrostatic potential surface of the VAT-1 dimer. (**b**) As in (**a**) but of the corresponding ribbon model.

The putative molecular function of VAT-1 has been discussed with respect to phospholipid binding^5^. Reported structures of lipid transfer and binding proteins reveal the presence of deep and long hydrophobic clefts or tunnels to accommodate long nonpolar hydrocarbon chains of lipids^20–23^. However, the VAT-1 dimer possesses no deep cleft or tunnel other than the nucleotide-binding site (Fig. 8). Although the nucleotide-binding site seems to be large enough to accommodate some phospholipids, the nature or mechanism of the binding specificity remains a serious question. The negatively charged head group of phospholipids may be trapped at the phosphate-binding site for NADP with some specificity. Further experimentation is required to clarify the potential lipid binding mechanism.

The Switch segment (Tyr285–Phe309) may be a specific feature for VAT-1 and its homologs, but not for other ζ-crystallin homologs such as Zta1 (Suppl. Fig. S8a). We found that the Switch segment participates in direct interactions with the bound NADP molecule, so that the function of the Switch segment should be coupled with nucleotide binding. In our structure, NADP binding induced folding of the Switch helix to facilitate protrusion of nonpolar residues such as tryptophan toward the outside, suggesting that NADP binding may trigger an intermolecular interaction with other binding partners. It is likely that the Switch helix may contribute to interaction with membranes, such as mitochondrial membranes to mediate recruitment of VAT-1 toward mitochondria.

We demonstrated oxidoreductase activity of VAT-1 in the presence of NADPH in vitro. The activity was observed with NQ and PQ, known substrates of Zta1 and ζ-crystallin^13,14^. In fact, the putative substrate binding site of VAT-1 resembles that of Zta1 and ζ-crystallin (Suppl. Fig. S8b). In addition, VAT-1 also displayed a substrate affinity towards PQ at a *K*_*m*_ value of 29.9 μM, which was comparable to those of the Zta1 (*K*_*m*_ = 4.56 μM^14^) and ζ-crystallin (*K*_*m*_ = 13 μM^13^) (Fig. 6b). The enzymatic studies further indicate VAT-1 is a NADPH-dependent quinone oxidoreductase. Since some reports indicated a putative function of VAT-1 in mitochondria dynamics, it is an interesting question whether quinone compounds participating in mitochondrial functions could act as substrates of VAT-1. However, we detected no activity against UQ. VAT-1 may contribute to the regulation of energy metabolism of cells by participating in some metabolic pathways but not in mitochondrial processes. The unknown authentic substrate(s) may be other quinone compounds such as cofactors essential for other processes in metabolic pathways. Further experiments are required to identify the substrate(s) and the role of the oxidoreductase activity of VAT-1.

## Methods

### Cloning, expression and purification

The cloned cDNA of human VAT-1 was purchase from GE Healthcare. Full-length (residues 1–393) and N-terminal truncated (residues 37–393 and 43–393) forms of the human VAT-1 cDNA fragments were amplified with KOD-Plus-PCR kit (TOYOBO), and with human VAT-1 cDNA as template DNA. PCR-amplified cDNA fragments were inserted between Sma I and Not I in pGEXM bacterial protein expression plasmid which has modified the multiple cloning site of pGEX 6P-3 (GE Healthcare) (Suppl. Fig. S9), with In-Fusion HD Cloning Kit (Clontech). The recombinant proteins are expressed as N-terminal glutathione *S*-transferase (GST)-fused proteins. The validity of the coding sequences were verified by DNA sequencing. *Escherichia coli* Rosetta 2 (DE3) (Novagen) was transformed with the plasmids. The cells were initially grown in a shaking incubator at 37 °C till the cell density reached an O.D. 0.6 at 595 nm. Then, the protein expression was induced by the addition of isopropyl-β-d-thiogalactoside (IPTG) to a final concentration of 100 µM following by incubation for 20 h at 16 °C for VAT-1 (43–393) or 20 °C for VAT-1 (full-length). The cells were harvested by centrifugation using Beckman Avanti HP 26XPI (4000 rpm for 15 min at 4 °C). Cells were then suspended in 2× PBS (phosphate-buffered saline) supplemented with 1 mM dithiothreitol (DTT) and 0.1% (v/v) Triton X-100 and disrupted by sonication using Qsonica (Q500) with 50% amplitude, 2 and 6 s on/off cycles for 45 min in an ice-bath. The soluble fraction was separated by ultracentrifugation using Beckman Optima XE-90 (30,000 rpm for 40 min at 4 °C). The supernatant containing the target protein was loaded onto a Glutathione Sepharose 4B resin column (GE Healthcare). The column was washed with buffer containing 20 mM Tris–HCl (pH 8.0), 100 mM NaCl and 1 mM DTT, and target protein was eluted using the same buffer containing 15 mM glutathione and cleaved using 4 units/ml HRV 3C protease (Merck) at 4 °C for overnight. The column effluent was loaded onto a HiTrap SP cation-exchange column (GE Healthcare) and eluted using a gradient of 0–500 mM NaCl in a buffer containing 25 mM MES (pH 6.0) and 1 mM DTT. The collected fraction was loaded onto a Superdex 200 gel filtration column (GE Healthcare) using buffer comprising 10 mM Tris–HCl (pH 7.2), 100 mM NaCl and 0.5 mM Tris(2-carboxyethyl)phosphine hydrochloride (TCEP). Analysis of the purified samples using SDS-PAGE (Suppl. Fig. S10) and matrix-assisted laser desorption/ionization time-of-flight mass spectrometry (MALDI-TOF MS; Bruker Daltonics) confirmed that the VAT-1 proteins (full-length and 43–393) were successfully purified without any degradation. The purified proteins were frozen in liquid nitrogen and stored at − 80 °C until use.

### Crystallization

Preliminary crystallization screening for both of the free- (full-length and 43–393) and NADP bound form were performed using the vapor-diffusion method at both 4 °C and 20 °C using commercially available screening kits (Hampton Research and QIAGEN). Proteins were mixed at 1:1 ratio with the reservoir solution. Crystals of free form of VAT-1 (43–393) were observed after 2 days in equilibrium against reservoir solution containing 100 mM BisTris (pH 6.0), 200 mM sodium nitrate and 22% (v/v) PEG 3350 (Hampton Research) at 20 °C. Crystals of VAT-1 (full-length) were found after 6 days in equilibrium against the solution containing 100 mM Tris–HCl (pH 7.0), 200 mM calcium acetate and 20% (v/v) PEG 3000 (QIAGEN) at 20 °C. The best crystal of VAT-1 (43–393) and NADP complex was obtained by the mixture of 1 µl protein–ligand solution (0.4 mM protein and 10 mM NADP in a buffer comprising 10 mM Tris–HCl (pH 7.2), 100 mM NaCl, 0.5 mM TCEP) and 1 µl reservoir solution [100 mM sodium citrate (pH 5.5) and 20% (v/v) PEG 3000 (Hampton Research)] after incubation for 3 days at 4 °C. Crystals were cryoprotected by the addition of 25% (v/v) glycerol for those of VAT-1 (full-length) and the NADP-bound VAT-1 (43–393). On the other hand, crystals of free form of VAT-1 (43–393) were cryoprotected in the solution containing 100 mM BisTris (pH 6.0), 200 mM sodium nitrate and 40% (v/v) PEG 3350. The cryoprotected crystals were flash-cooled using liquid nitrogen until use.

### X-ray data collection, phasing and refinement

All X-ray data were collected at SPring-8, Harima, Japan. During X-ray beam exposure, crystals were flash-cooled and maintained at 100 K using a nitrogen stream. Detailed statistics of the structure determination are shown in Supplementary Table S1. All diffraction data were indexed and merged using the XDS program^24^. Molecular replacement was successfully performed using the PHASER program^25^ in Phenix^26^, and the structure of VAT-1 homolog-like protein (PDB ID 4A27), which displays 54% sequence identity, as a reference model. The built model was refined through alternating cycles using the Coot^27,28^ and phenix.refine programs^29^. Superposition of VAT-1 was performed using the program Superpose in Phenix^26^. Comparison of our VAT-1 to the structures in PDB was performed with MATRAS server^30^. The alignment of the amino acid sequences were performed using ClustalOmega^31^. Illustrations were prepared using the program PyMOL Molecular Graphics System, Version 1.5.0.3 Schrödinger, LLC. The cavity calculations were performed using Caver 3.0.3 PyMol plugin^32^. The interaction of VAT-1 and NADP was calculated by LIGPLOT^33^.

### Size exclusion chromatography (SEC)

Analytical gel filtration was performed with VAT-1 (full-length) and VAT-1 (43–393) in the absence or presence of NADP by loading onto an analytical gel filtration column, Superdex 200 (10/30) (GE Healthcare), in buffer containing 10 mM Tris–HCl (pH 7.2), 100 mM NaCl and 0.5 mM TCEP at 8 °C. In the absence of NADP, total volume 250 µl of VAT-1 protein samples were loaded onto the column at concentrations of 10 µM, 20 µM, 50 µM, and 300 µM. For the analysis of protein–ligand complex, various concentrations of VAT-1 proteins (10 µM, 20 µM, and 50 µM) were incubated with NADP (10-folds higher molar concentrations) at 8 °C for 2 h prior to gel-filtration. The mixtures were then loaded onto the same analytical gel filtration column under the same condition as previously mentioned. The elution profiles were compared with that of Gel Filtration Standards (Bio-Rad). The molecular weights of the eluted peaks were estimated using monomeric standard molecular weights, where VAT-1 (full-length) weighted 42,074 Da and VAT-1 (43–393) weighted 38,013 Da.

### Binding assay using calorimetry

Binding studies utilizing isothermal titration calorimetry (ITC) were conducted using a calorimeter (MicroCal iTC200, GE Healthcare) at 20 °C. Purified proteins were dialyzed overnight in buffer containing 10 mM HEPES (pH 7.0), 100 mM NaCl and 0.1 mM TCEP. NADPH solution (2 mM) was injected (0.5 μl each, 3 min pause) into VAT-1 (full-length) protein solution (0.1 mM). Details of each titration are described in the figure legends of Supplementary Fig. S2. Data fitting was performed using a 1:1 binding model (n = 1) of the ORIGIN software program (Origin version 7.0, OriginLab Corporation, Northampton, MA, USA) supplied with the instrument and low-c ITC method^16^ was used for measuring the weak interaction between VAT-1 and NADPH.

### Enzymatic activity assay

Enzymatic activity was investigated by monitoring the decrease in NADPH absorption at 340 nm. All assays were performed at 25 °C in a standard assay mixture containing 100 mM Tris–HCl (pH 7.8), 100 μM NADPH, 0.5 μM VAT-1 (full-length/ 43–393) and substrates at various concentrations. The quinone substrates used were 1,2-naphthoquinone (#346616, Sigma), 9,10-phenanthrenequinone (#156507, Sigma), Menadione (#M5625, Sigma), and coenzyme Q_0_ (#D9150, Sigma). All quinone substrates were dissolved in ethanol, with the final concentration of alcohol in the assay being 2.5%. Reactions were initiated by the addition of VAT-1, and the decrease in absorbance at 340 nm (ε_(NADPH)_ = 6220 M^−1^ cm^−1^) was monitored using a UV-1900i spectrophotometer equipped with a temperature-controlled cuvette holder TCC-100 (Shimadzu). The decrease in absorbance resulting from the non-enzymatic reduction of quinone substrates upon addition of NADPH was set as the background control for each assay. All reactions were measured in triplicates.

## Supplementary Information

Supplementary information.

## Data Availability

*Accession code* Protein Data Bank: The atomic coordinates and structure factors for the reported crystal structures of human VAT-1 (43–393) are deposited under accession codes 6LII (free form) and 6LHR (NADP-bound form).
