# Supplementary material for: Structural insights into vesicle amine transport-1 (VAT-1) as a member of the NADPH-dependent quinone oxidoreductase family
Source: Sci Rep. 2021 Jan 22;11:2120. doi: 10.1038/s41598-021-81409-y (PMC7822847; doi:10.1038/s41598-021-81409-y)
Supplement: Supplementary file 1 — Supplementary information. [file 41598_2021_81409_MOESM1_ESM.pdf]

Structural insights into Vesicle Amine Transport-1（VAT-1）as a member of the NADPH-dependent quinone oxidoreductase family

Sun-Yong Kim[1,3], Tomoyuki Mori[1,3], Min Fey Chek[1,3], Shunji Furuya[1], Ken Matsumoto[2], Taisei Yajima[2], Toshihiko Ogura[2] and Toshio Hakoshima[1]*

[1] Structural Biology Laboratory, Nara Institute of Science and Technology, 8916-5 Takayama, Ikoma, Nara 630-0192, Japan
[2] Department of Developmental Neurobiology, Institute of Development, Aging and Cancer, Tohoku University, 4-1 Seiryo, Aoba, Sendai, Miyagi 980-8575, Japan.
[3] These authors contributed equally: Sun-Yong Kim, Tomoyuki Mori, Min Fey Chek
* Corresponding author:
T. Hakoshima, Structural Biology Laboratory, Nara Institute of Science and Technology,
8916-5 Takayama, Ikoma, Nara 630-0192, Japan.
Tel.: +81-743-72-5570; Fax: +81-743-72-5579; E-mail: hakosima@bs.naist.jp

Supplementary Figures

Supplementary Table 1 | Crystallographic statistics of the human VAT-1(43-393)-NADP complex and free form.

Supplementary Figure 1 | Sequence alignment of VAT-1.
Supplementary Figure 2 | Microcalorimetry of NADP titration into VAT-1.
Supplementary Figure 3 | Structural comparison of the crystallographic independent VAT-1 molecules in the crystals.
Supplementary Figure 4 | Structural comparison of VAT-1 and the related proteins of the oxidoreductase superfamily.
Supplementary Figure 5 | Accessible entrance and exit of the nucleotide-binding tunnel.
Supplementary Figure 6 | Crystal packing shows direct contacts between Switch segments
Supplementary Figure 7 | Size exclusion chromatography of VAT-1.
Supplementary Figure 8 | Structural comparison between VAT-1 and Zta1 in the NADP-bound states.
Supplementary Figure 9 | Plasmid for the VAT-1 expression system
Supplementary Figure 10 | SDS PAGE analysis of purified VAT-1 (Full-length) and VAT-1 (43-393)

Supplementary Table 1 | Crystallographic statistics of the human VAT-1(43-393)-NADP complex and free form.

Samples	Human VAT-1(43-393)-NADP complex	Human VAT-1(43-393) free form
**Data collection**		
X-ray source	BL41XU	
Detector	EIGER 16M	
Wave length	1 Å	
Oscillation range	0 ~ 180 ˚ (0.1 ˚ X 1800 images)	
**X-ray data (XDS)**		
Space group	*C*2	$P3_221$
Cell parameter [Å]	*a*=176.3, *b*=96.3, *c*=112.4	*a*=69.3, *b*=69.3, *c*=581.8
	α=γ=90 ˚, β=123.1 ˚	α=β=90 ˚, γ=120 ˚
Resolution [Å]	50 ~ 2.62 (2.79 ~ 2.62)	50 ~ 2.30 (2.44 ~ 2.30)
Reflections, total/unique	155597/46822	707889/74674
Completeness [%]	98.5 (98.5)	99.9 (99.3)
Redundancy	3.3 (3.4)	9.5 (8.8)
$<I/\sigma_I>$	10.0 (2.1)	13.3 (2.4)
R_{merge}[%]	9.5 (64.0)	10.9 (57.5)
R_{meas} [%]	11.4 (76.1)	11.5 (61.1)
$CC_{1/2}$ [%]	99.5 (83.9)	99.8 (73.1)
**Refinement**		
Resolution [Å]	48.15 ~ 2.62 (2.68 ~ 2.62)	48.7 ~ 2.30 (2.34 ~ 2.30)
R_{work}/R_{free} [%]	20.8/24.6 (31.46/35.29)	18.0/20.3 (27.7/28.8)
Mean *B* value		
protein	69.3	45.2
ligands (NADP)	62.5	-
waters	60.2	37.9
Wilson *B* factor	63.7	45.3
Ramachandran plot [%]		
favored	97.5	98.0
allowed	2.5	2.0
outliers	0	0
Rotamer outliers	3.8	0
C-beta outliers	0	0
Clashscore	7.9	8.5
Basic geometry [rmsd]		
bond length [Å]	0.008	0.006
bond angle [˚]	0.955	0.677

Each data set was collected using single crystal. R_{free} was calculated with 5% reflections of the data. Highest resolution shell is represented in parentheses.

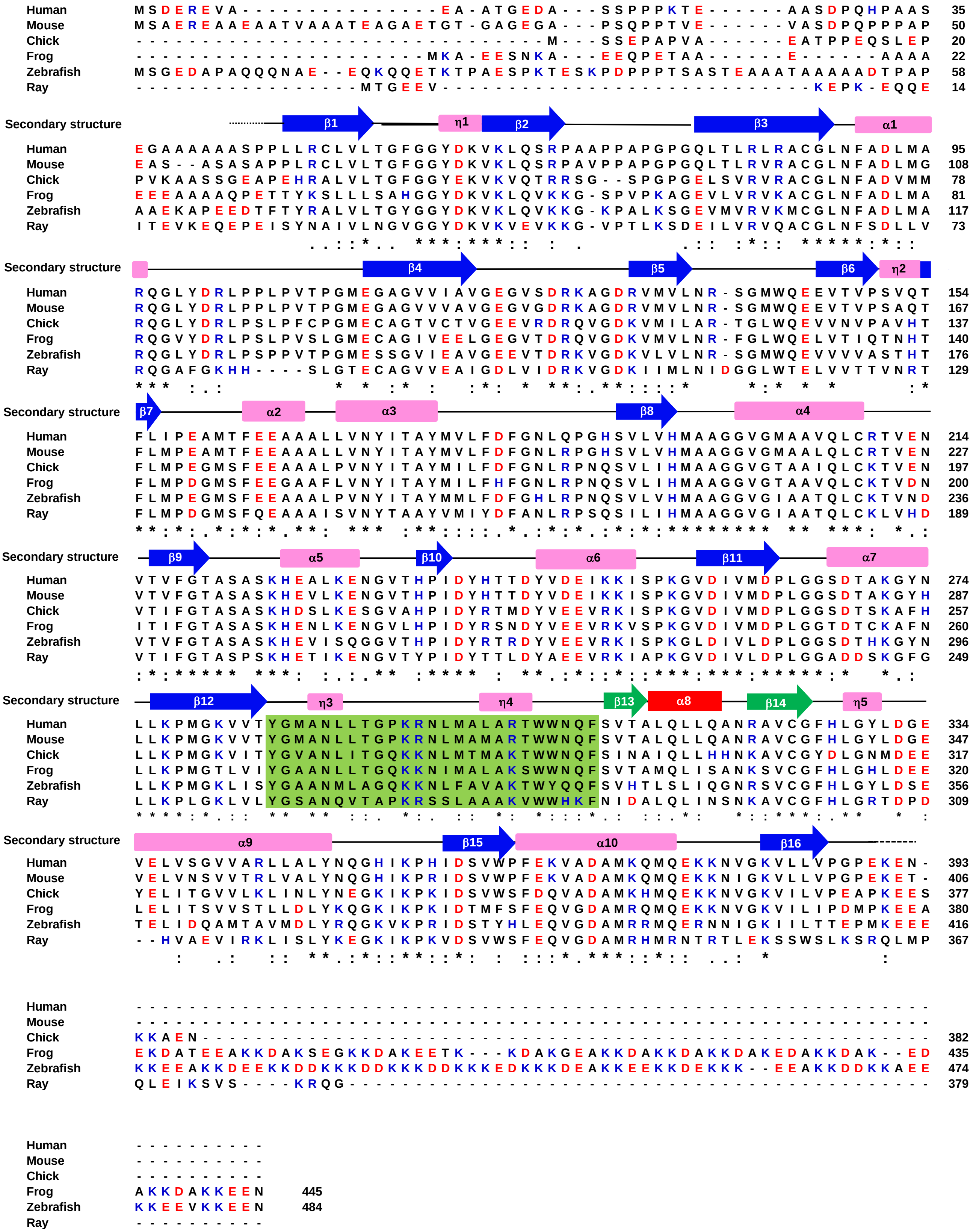

Supplementary Figure 1 | Sequence alignment of VAT-1.

Amino acid sequence alignment of vertebrate VAT-1 proteins; human: *Homo sapiens* (RefSeq code: NM_006373.4), mouse: *Mus musculus* (RefSeq code: NP_036167.1), chick: *Gallus gallus* (RefSeq code: XP_418130.5), Frog: *Xenopus laevis* (RefSeq code: NP_001085972.1), zebrafish: *Danio rerio* (RefSeq code: NP_001007052.3) and ray: *Torpedo californica* (UniProtKB/Swiss-Prot code: P19333.1), with Clustal Omega. Sequence identity values of mouse, chick, frog, zebrafish and ray VAT-1 compared with human VAT-1 are 90.0, 67.3, 64.6, 61.7 and 49.3%, respectively. Acidic (Glu and Asp) and basic (Arg, Lys and His) residues are colored red and blue, respectively. The Switch segment is enclosed within a green box. The secondary structure, based on the human VAT-1 (43-393) structure determined in this study, is shown over the sequence alignment as pink boxes (α-helices and 3_{10} helices) and blue arrows (β-strands). The secondary structure involved in dimerization of VAT-1 is shown as green arrows (β13 and β14) and a red box (α8).

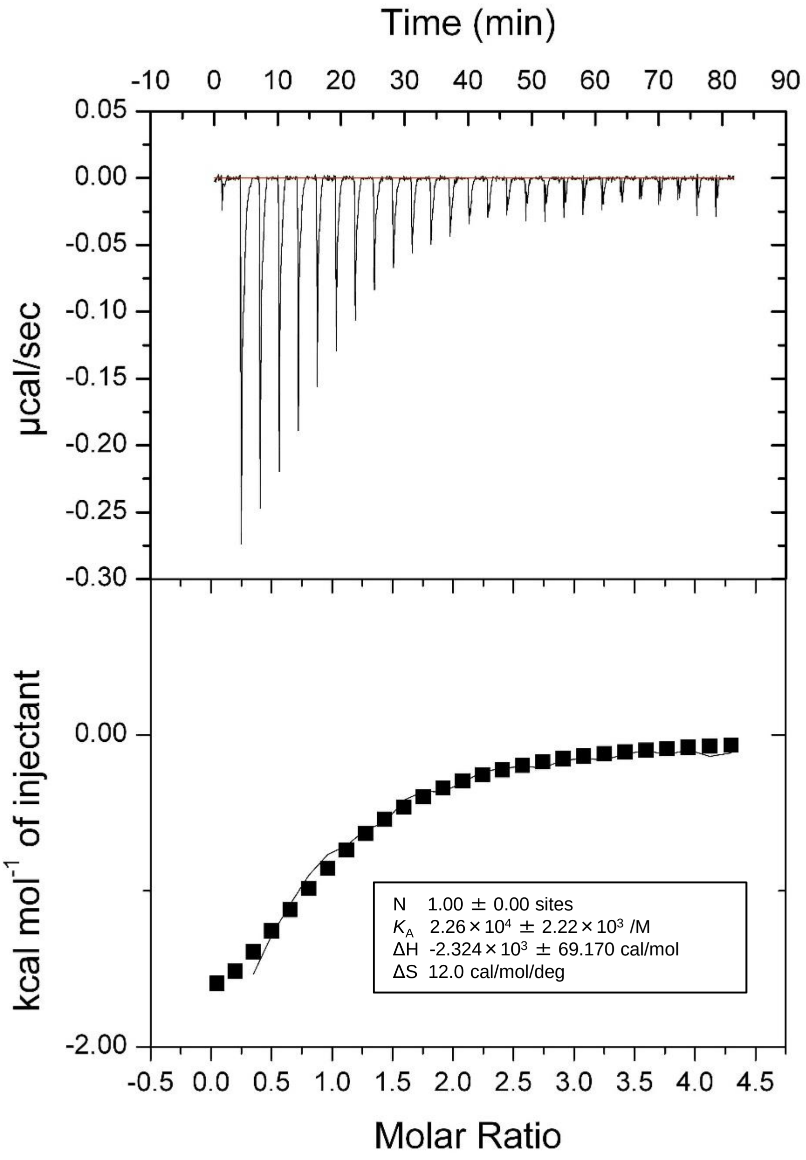

Supplementary Figure 2 | Microcalorimetry of NADPH titration into VAT-1

Isothermal titration calorimetry (ITC) profile for binding of NADPH to VAT-1 (Full-length). Raw data for 27 sequential injections (the upper panel) and the plot of the heat evolved (kcal) per mole of NADPH added, corrected for the heat of NADPH by dilution, against the molar ratio of NADPH to the VAT-1. The data except for the first trial injection were analyzed.

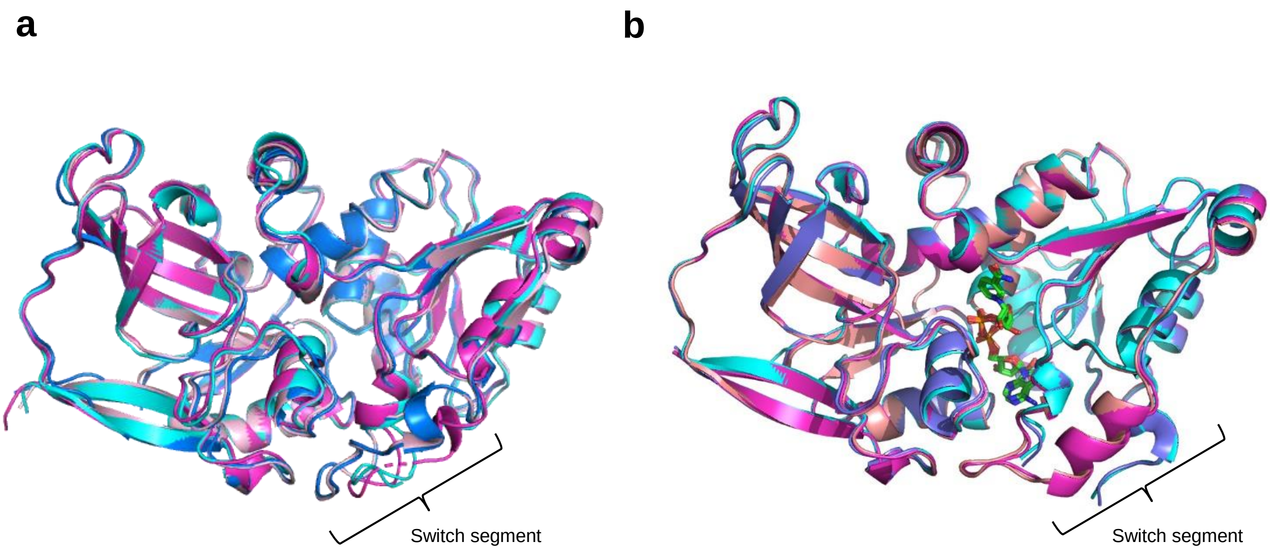

Supplementary Figure 3 | Structure comparison of the crystallographically independent VAT-1 molecules in the crystals.

a, Four crystallographically independent molecules (A-D) of the free form display essentially the same structure with local conformational changes at the Switch segment. The root-mean-square (rms) deviation between molecules A and C and between B and D is small (0.4-0.5 Å), whereas that between the pairs is relatively large (1.3-1.4 Å).

b, Four crystallographically independent molecules of the NADP-bound form display essentially the same structure with local conformational changes at the Switch segment.

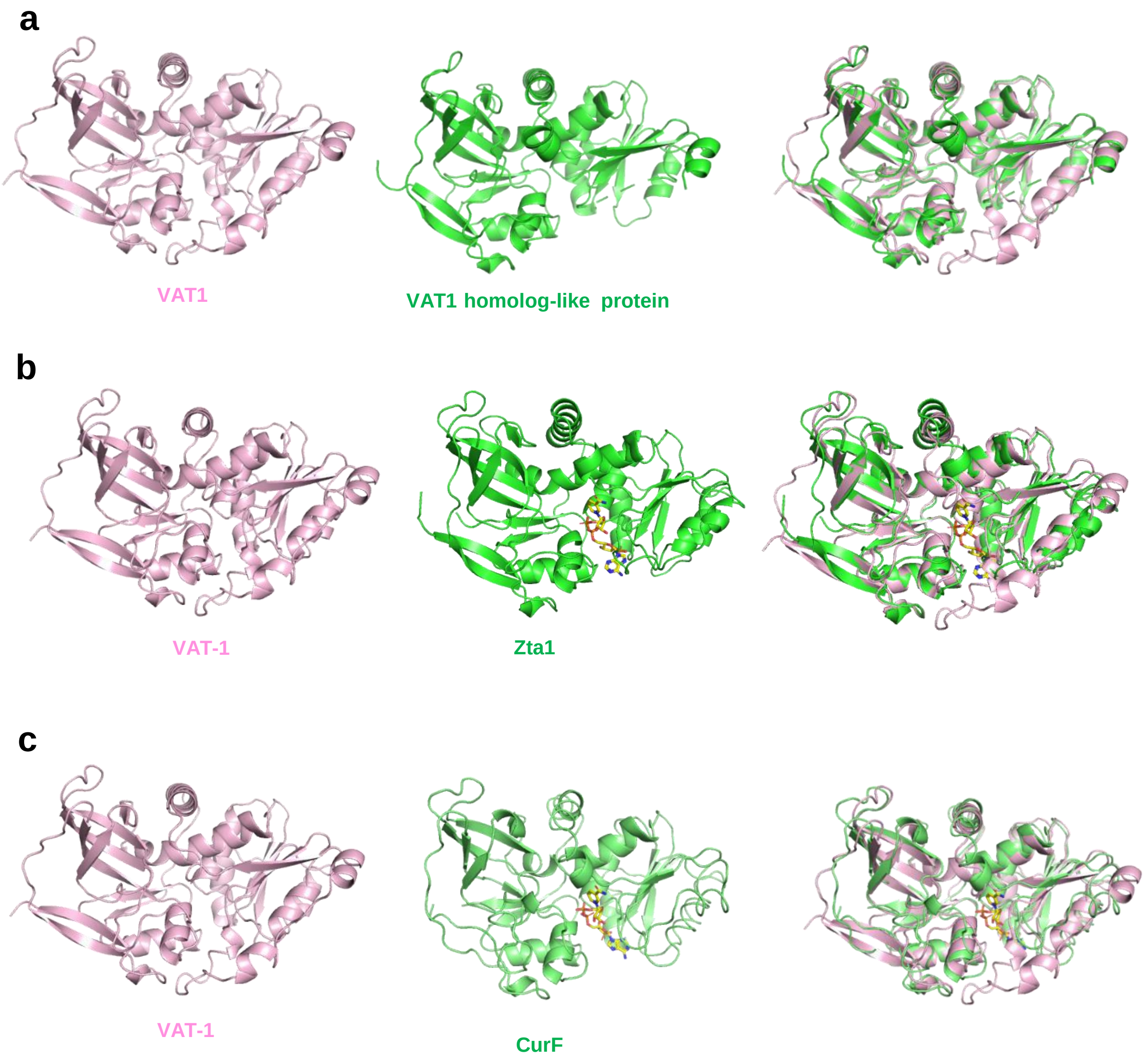

Supplementary Figure 4 | Structural comparison of VAT-1 and related proteins of the oxidoreductase superfamily.
a, Human VAT-1 and human VAT-1 homolog-like protein (PDB ID 4a27), which exhibits 45.8% sequence identity and root-mean-square (rms) deviation of 1.13 Å for Cα atoms.
b, Human VAT-1 and *Saccharomyces cerevisiae* ζ-crystallin-like quinone oxidoreductase Zta1 (3qwb), which exhibits 25.5% sequence identity and rms deviation of 1.51 Å for Cα atoms. The NADP molecule bound to Zta1 is shown as a stick model (color codes: C in yellow, N in blue, O in red and P in orange).
c, Human VAT-1 and *Lyngbya majuscule* CurF ER (5dp2), which exhibits 26.9% sequence identity and rms deviation of 1.67 Å for Cα atoms. The NADP molecule bound to CurF is shown as a stick model (color codes as in **b**).

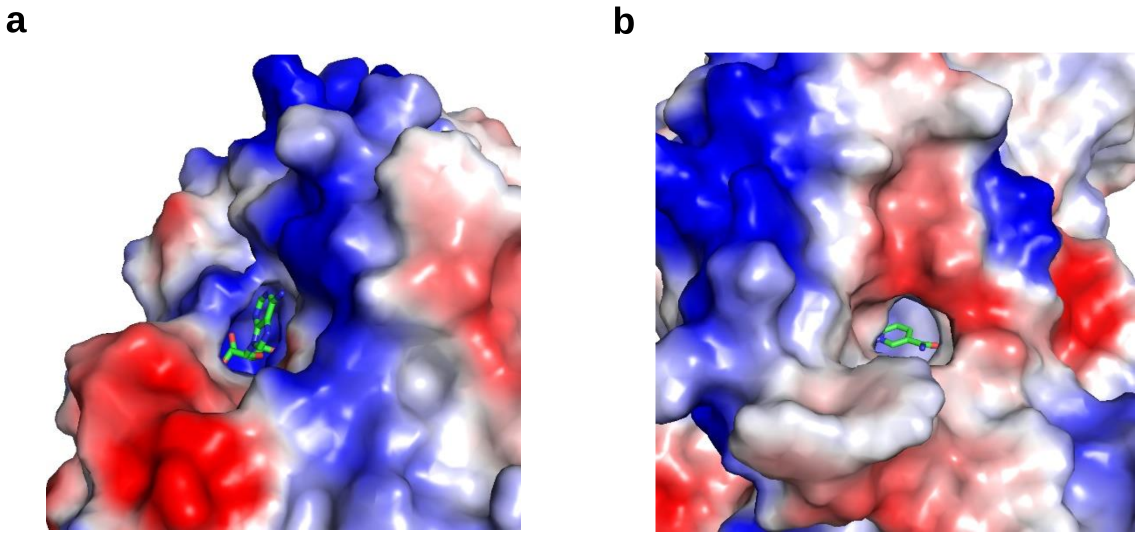

Supplementary Figure 5 | Close up view of accessible entrance and exit of the nucleotide-binding tunnel.
VAT-1 structure is represented with the protein contact potential calculated by PyMOL. Amino and carboxyl groups in the surface are colored in blue and red respectively. NADP molecule is shown as stick model. **a**, A small accessible hole of the ADP-binding site. The adenine ring of the bound NADP molecule is seen in the hole. **b**, A small accessible hole of the NMN-binding site. The nicotinamide moiety of the bound NADP molecule is seen in the hole.

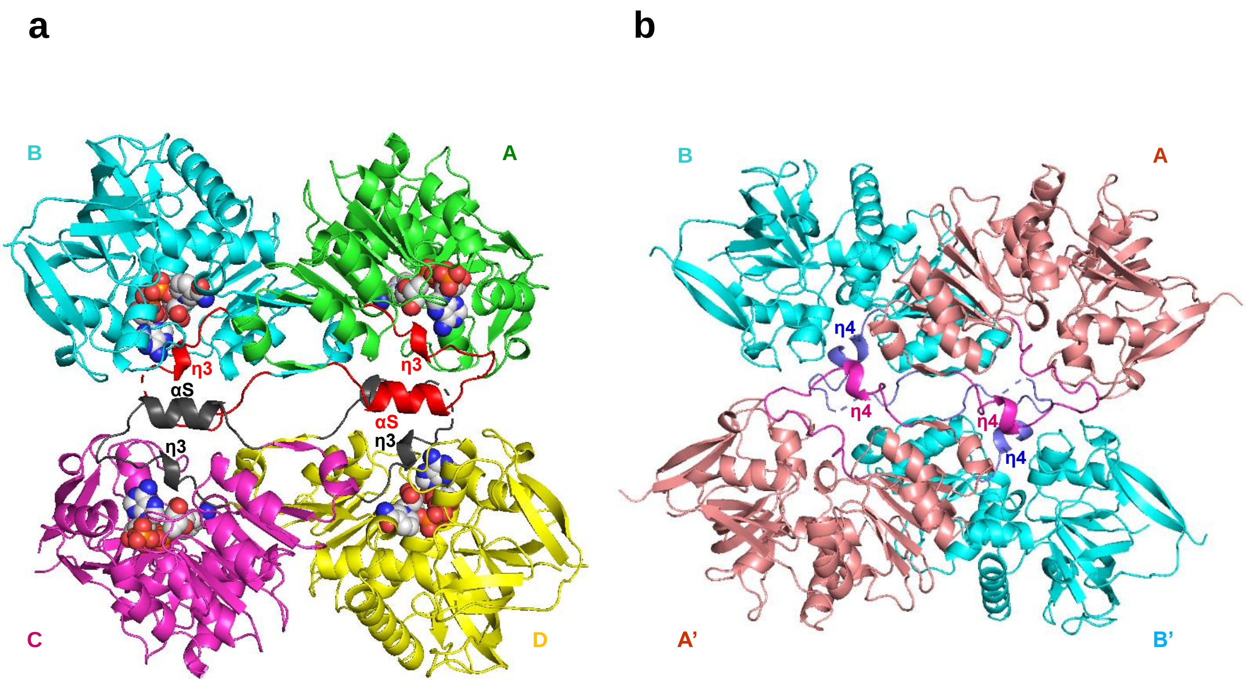

Supplementary Figure 6 | Crystal packing shows direct contacts between Switch segments.

a, VAT-1 in the NADP-bound form. Molecules A (green) and B (cyan) form a homodimer and contact with the other homodimer of molecules C (magenta) and D (yellow) at the Switch segments. The Switch segments of molecules A and B are in red, while those of molecules C and D are in black. The Switch segment of molecule A makes nonpolar contacts with that of molecule D, while the Switch segment of molecule B makes nonpolar contacts with that of molecule C. The Switch segments of molecules B and D are partially disordered. The core of the nonpolar contacts is formed by hydrophobic contacts between nonpolar residues, including Trp305 and Trp306, from αS-helices. The NADP molecules are shown as space-filling models (carbon in grey, nitrogen in blue and in red and phosphorus in orange).

b, VAT-1 in the free form. Molecules A (orange) and B (cyan) form a homodimer and make contact with symmetry-related molecules A’ and B’ at the Switch segments. Molecule A contacts B’, while molecule B contacts A’. The Switch segments of molecules A and A’ (magenta) make nonpolar contacts with those of molecules B’ and B (blue). Molecule B displays a partially disordered Switch segment. Molecules C and D display similar modes of crystal contacts. The core of the nonpolar contacts is formed by hydrophobic contacts between nonpolar residues, including Trp305 and Trp306, from η4-helices.

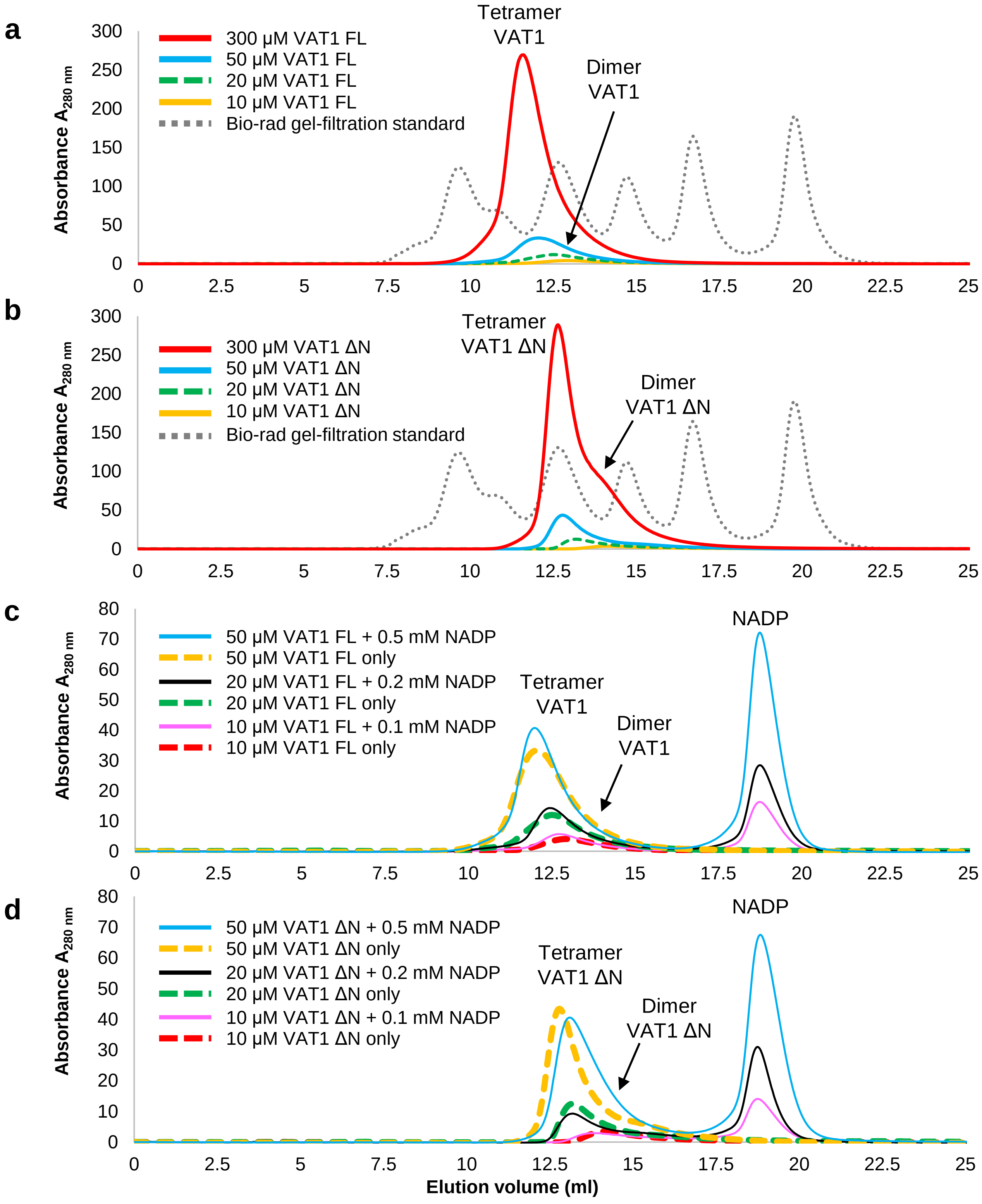

Supplementary Figure 7 | Size exclusion chromatography of free form VAT-1 (Full-length) and VAT-1 (43-393).
Size exclusion chromatography (SEC) was performed using a Superdex 200 10/30 column with buffer: 10 mM Tris-HCl (pH 7.2), 100 mM NaCl, 0.5 mM TCEP at 8˚C.
(**a**) SEC analysis of free form VAT-1 (full-length, 42 kDa) at the concentrations of 10, 20, 50, and 300 μM.
VAT-1 (full-length) exists as dimer at 10 μM, and shifted to tetramer as concentrations increased. The bio-rad gel-filtration standard (dotted lines) corresponds to 670 kDa (peak 1), 158 kDa (peak 2), 44 kDa (peak 3), 17 kDa (peak 4), and 1375 Da (peak 5).
(**b**) Same as (a), but VAT-1 (43-393) was employed. VAT-1 (43-393) exists as dimer at 10 μM and co-exists as dimer-tetramer equilibrium at higher concentrations.
(**c**) The oligomerization of 10, 20, and 50 μM VAT-1 (full-length) in-solution was compared in the presence of NADP (10-fold higher concentrations). The mixture was incubated for 2 hours at 8˚C prior to SEC analysis. The oligomerization of the full-length VAT-1 was unaffected by the addition of NADP.
(**d**) Same as (a), but VAT-1 (43-393) was employed. In the presence of 500 μM NADP, VAT1 (43-393) exists in dimer-tetramer equilibrium (skyblue line), whereas full-length VAT-1 did not displayed any difference with or without the presence of NADP.

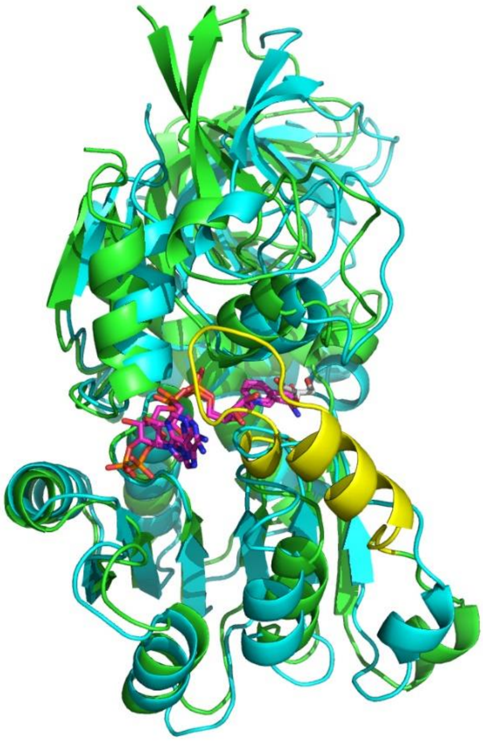

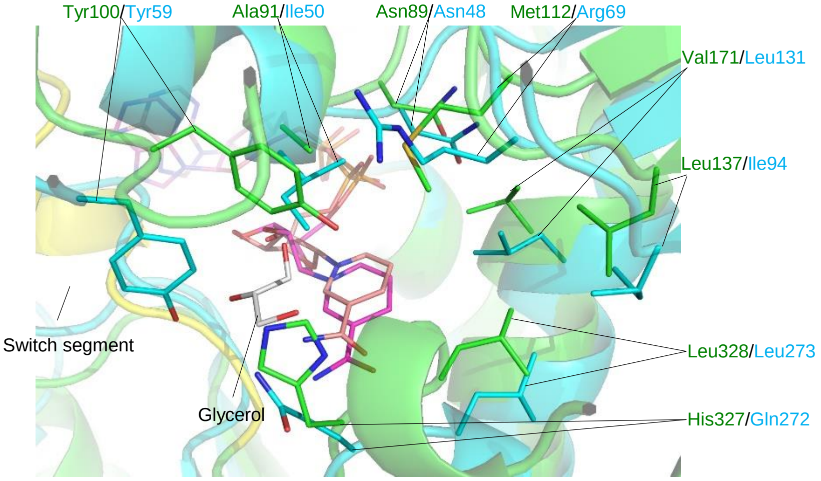

Supplementary Figure 8 | Structural comparison between VAT-1 and Zta1 in the NADP-bound states.
a, Structural overlay of NADP-bound VAT-1 (green) onto NADP-bound Zta1 (cyan). VAT-1 possesses a long Switch segment (yellow) covering the nucleotide-binding site. The bound NADP molecules are shown as stick models (color codes: C in magenta, N in blue, O in red and P in orange).
b, Structural overlay of the putative substrate-binding sites found in NADP-bound VAT-1 (green) onto NADP-bound Zta1 (cyan). VAT-1 possesses a long Switch segment (yellow) covering the nucleotide-binding site. The bound NADP molecules are shown as stick models (color codes: C in magenta, N in blue, O in red and P in orange). A glycerol molecule (C in grey) is found in the binding site of Zta1.

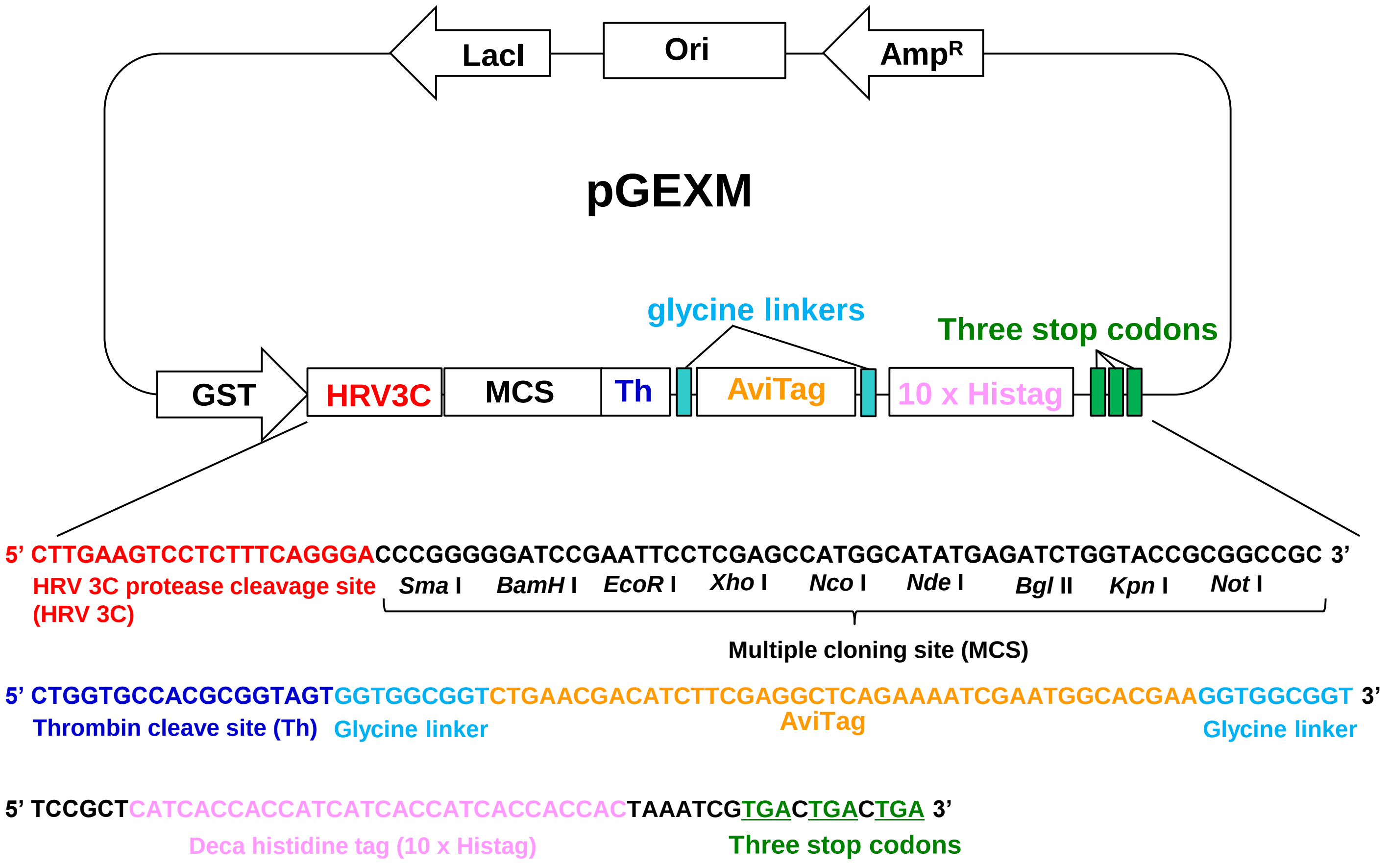

Supplementary Figure 9 | Plasmid for the VAT-1 expression system

Representation of the plasmid map of pGEX 6P-3 (GenBank code: U78874.1) modified at the multiple cloning site (MCS). The modified pGEX 6P-3 plasmid, named pGEXM, is used as a bacterial expression plasmid for VAT-1 in this study. The pGEXM has a DNA linker between the HRV 3C protease cleavage site and three stop codons originating from pGEX6P-3. In the pGEXM, a lac repressor gene (Lac I) , a pBR322 origin (Ori), a β-lactamase gene (Amp^R) and a glutathione-*S*-transferase (GST) gene are shown as a box and arrows, respectively. The DNA linker sequence is shown under the plasmid map. The linker has a human rhinovirus (HRV) 3C protease-cording sequence (red), a multiple cloning site containing restriction enzyme cleave sequences of *Sma* I, *Bam*H I, *Eco*R I, *Xho* I, *Nco* I, *Nde* I, *Bgl* II, *Kpn* I and *Not* I, a thrombin cleave site (blue), two glycine linkers (cyan), an AviTag-cording sequence (orange), deca histidine tag-cording sequence (pink), and three stop codons (green). The cDNA regions cording 1-393, 37-393 and 43-393 of amino acid residues of VAT-1 are cloned into the pGEXM between *Sma* I and *Not* I restriction enzyme site with a stop codon.

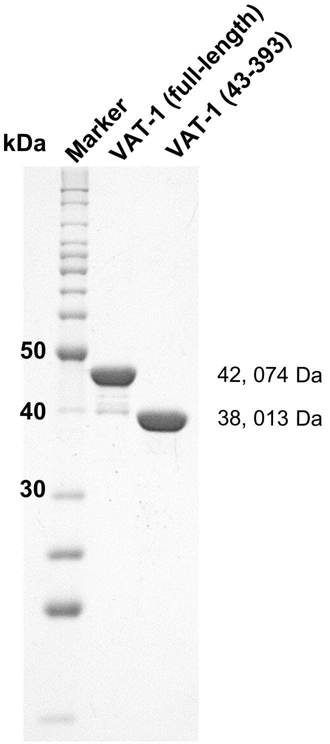

Supplementary Figure 10 | SDS PAGE analysis of purified VAT-1 (Full-length) and VAT-1 (43-393)
Purified VAT-1 (full-length) and VAT-1 (43-393) (2 μg) were shown in a SDS-PAGE analysis.
BenchMark™ Protein Ladder (Invitrogen) was used as reference to the molecular weight of target proteins.
The observed molecular weight of both VAT-1 constructs were in agreement with the expected molecular weight of VAT-1 (full-length) at 42 kDa, and VAT-1 (43-393) at 38 kDa.
